# Mechanical behavior of hybrid glenoid components compared to all-PE components: a finite element analysis

**DOI:** 10.1186/s40634-022-00494-8

**Published:** 2022-06-19

**Authors:** Nicolas Bonnevialle, Julien Berhouet, Paul Pôtel, Jacobus Hendrik Müller, Shoulder Friends Institute, Arnaud Godenèche

**Affiliations:** 1 Hôpital Pierre Paul Riquet - CHU de Toulouse - Place Baylac, 31059 Toulouse Cedex 09, France; 2 Faculté de Médecine de Tours - CHRU Trousseau Service d’Orthopédie Traumatologie, Université de Tours, 1C Avenue de la République, 37170 Chambray-les-Tours, France; 3 Laboratoire d’Informatique Fondamentale et Appliquée de Tours EA6300, Equipe Reconnaissance de Forme Et Analyse de L’Image, Université de Tours – Ecole d’Ingénieurs Polytechnique Universitaire de Tours, 64 Avenue Portalis, 37200 Tours, France; 4 MOVE-UP SAS, Alixan, France; 5 ReSurg S.A, Chemin de la Vuarpillière 35, 1260 Nyon, Switzerland; 6 Shoulder Friends Institute, Paris, France; 7 Centre Orthopédique Santy, Ramsay Santé, Hôpital Privé Jean Memoz, Lyon, France

**Keywords:** Total shoulder arthroplasty, Hybrid glenoid component, Micromotion, Stress, Finite element analysis

## Abstract

**Purpose:**

The purpose of this finite element study was to compare bone and cement stresses and implant micromotions among all-polyethylene (PE) and hybrid glenoid components. The hypothesis was that, compared to all-PE components, hybrid components yield lower bone and cement stresses with smaller micromotions.

**Methods:**

Implant micromotions and cement and bone stresses were compared among 4 all PE (U-PG, U-KG, A-KG, I-KG) and 2 hybrid (E-hCG, I-hPG) virtually implanted glenoid components. Glenohumeral joint reaction forces were applied at five loading regions (central, anterior, posterior, superior and inferior). Implant failure was assumed if glenoid micromotion exceeded 75 µm or cement stresses exceeded 4 MPa. The critical cement volume (CCV) was based on the percentage of cement volume that exceeded 4 MPa. Results were pooled and summarized in boxplots, and differences evaluated using pairwise Wilcoxon Rank Sum tests.

**Results:**

Differences in cement stress were found only between the I-hPG hybrid component (2.9 ± 1.0 MPa) and all-PE keeled-components (U-KG: 3.8 ± 0.9 MPa, *p* = 0.017; A-KG: 3.6 ± 0.5 MPa, *p* = 0.014; I-KG: 3.6 ± 0.6 MPa, *p* = 0.040). There were no differences in cortical and trabecular bone stresses among glenoid components. The E-hCG hybrid component exceeded micromotions of 75 µm in 2 patients. There were no differences in %CCV among glenoid components.

**Conclusions:**

Finite element analyses reveal that compared to all-PE glenoid components, hybrid components yield similar average stresses within bone and cement. Finally, risk of fatigue failure of the cement mantle is equal for hybrid and all-PE components, as no difference in %CCV was observed.

**Level of evidence:**

IV, in-silico.

**Supplementary Information:**

The online version contains supplementary material available at 10.1186/s40634-022-00494-8.

## Introduction

Total shoulder arthroplasty (TSA) is an effective and reliable treatment for shoulder arthritis [1, 2]. Among other factors, its success relies on stable glenoid fixation [3], either using cemented all-polyethylene (PE) components, or uncemented porous metal-backed PE components. To grant stability, the bone-implant interface of glenoid components may feature a keel, a peg, or a combination of both.

Despite numerous attempts to improve fixation of TSA glenoid components [4], glenoid loosening remains a frequent cause of failure [1, 5, 6]. Most failures of all-PE components are attributed to debris caused by micro-cracks in the cement mantle due to fatigue, whereas failures of porous metal-backed PE components are attributed to micromotion exceeding thresholds that inhibit bony in-growth [7]. According to a recent review of joint registry data [8], metal-backed PE components have higher risks of revision, compared to cemented all-PE components.

Hybrid glenoid components, featuring porous metal or metal-coated pegs on a PE bone-implant interface, were recently introduced to combine the benefits of cemented all-PE and uncemented metal-backed PE components [2]. Hybrid components aim to enhance initial fixation with a cemented PE surface, which reduces micromotions and thereby promotes bony in-growth within the uncemented porous pegs to grant long-term biologic fixation [5, 9]. Therefore, the purpose of this finite element study was to compare bone and cement stresses and implant micromotions among all-PE and hybrid components. The hypothesis was that, compared to all-PE components, hybrid components yield lower stresses within the bone and cement, and have smaller micromotions.

## Material and methods

### Anatomic models

Micromotions and stresses within bone and cement were compared among glenoid components that were virtually implanted in scapulae of three men and two women scheduled for shoulder arthroplasty (Table 1) [10]. The patients had provided written informed consent for the use of their data and images for research and publishing purposes and the institutional review board approved the study in advance (IRB reference number: COS-RGDS-2021–05-004-GODENECHE-A). Computed tomography (CT) scans (SOMATOM Definition AS, Siemens Healthcare SAS, France) with standardized scanning parameters (281 mA; 120kVp; B31s reconstruction kernel) of the 5 shoulders were segmented (VolView ver. 3.2, Kitware, Clifton Park, NY, USA), by manually separating the scapula from the clavicle along the acromioclavicular joint. For all scapulae, the boundary between cortical and trabecular bone was differentiated in each slice, using the thresholding (trabecular bone < 20% of the maximum density [10]) and manual selection tools of the segmentation software. The bone density in each voxel was estimated by applying the method described by Pomwenger et al. [11] (Additional file 1). Mean cortical (${\overline{\rho }}_{cortical}$) and mean trabecular (${\overline{\rho }}_{trabecular}$) densities were first calculated, before the Young’s moduli ($E$) were assigned to the cortical and trabecular bone volumes according to the method proposed by Pomwenger et al. [12] (Table 1). A Poisson ratio of 0.3 was assigned for both cortical and trabecular bone [13].

**Table 1:** Patient characteristics

	**Patient**				
	**P1**	**P2**	**P3**	**P4**	**P5**
**Anatomical parameters**					
Sex	Man	Woman	Man	Woman	Man
Side	Right	Right	Left	Left	Right
Age (years)	41	49	40	41	46
Height (cm)	193	163	170	168	182
Weight (kg)	105	47	85	74	98
BMI	28	18	29	26	29
Retroversion (°)	3	4	0	4	5
Inclination (°)	2	9	7	13	4
**Simulation parameters**					
Glenoid diameter (mm)	70.2	60	68.8	70.6	68.2
Cortical Young's Modulus (MPa)	4336	2633	1880	4259	2617
Trabecular Young's Modulus (MPa)	305	33	43	68	144
Joint reaction force (N)	903	404	729	636	839
* Mesh density (number of elements)*					
Cortical bone	189 000	114 000	157 000	152 000	144 000
Trabecular bone	33 000	43 000	12 000	52 000	10 000

Abbreviations: *P* patient, *BMI* body mass index

The segmented geometries were then Imported into SolidWorks 2016 (Dassault Systèmes, SolidWorks Corporation, Waltham, MA) for further processing. 

### Implant configurations

**Fig. 1:**
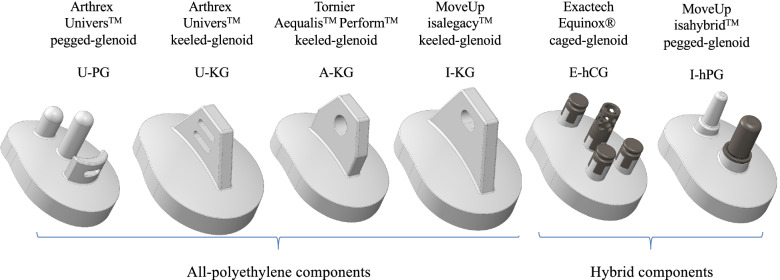
Illustration of the 6 glenoid components of which 4 are all-PE and 2 are hybrid designs

Six three-dimensional (3D) computer assisted design (CAD) glenoid component models were created using SolidWorks 2016 (Dassault Systèmes, SolidWorks Corporation, Waltham, MA) (Table 2, Fig. 1). Measurements were obtained from the manufacturer, and where unavailable, physically measured on component explants. Four of the 6 glenoid components were all-PE designs and 2 were hybrid designs. Of the 4 all-PE components, 1 (U-PG) had two pegs in combination with a keel, and 3 (U-KG, A-KG, I-KG) had a keel. Of the 2 hybrid components, 1 (E-hCG) had a porous titanium “cage” in combination with three titanium pegs, and 1 (I-hPG) had a porous titanium peg in combination with a PE peg.

**Table 2:** TSA glenoid baseplate designation and properties

	**All-polyethylene components**				**Hybrid components**	
	**Arthrex** **Univers™** **pegged-glenoid**	**Arthrex** **Univers™** **keeled-glenoid**	**Tornier** **Aequalis™ Perform™** **keeled-glenoid**	**MoveUP** **isalegacy™** **keeled-glenoid**	**Exactech** **Equinox®** **caged-glenoid**	**MoveUP** **isahybrid™** **pegged-glenoid**
**Gelnoid parameters**						
Designation	U-PG	U-KG	A-KG	I-KG	E-hCG	I-hPG
Baseplate/bone interface	2 PE pegs & 1 PE keel	1 PE keel	1 PE keel	1 PE keel	1 Porous Ti cage & 3 Ti pegs	1 Porous Ti peg & 1 PE peg
Fixation	All cemented	All cemented	All cemented	All cemented	All cemented except Ti cage	All cemented except Ti peg
* Glenoid size used in the anatomical models*						
Patient 1	x-Large	x-Large	XL50	Size 4 reaming Ø80 mm	Beta, Extra Large	Size 4 reaming Ø80 mm
Patient 2	Small	Small	M30	Size 2 reaming Ø60 mm	Alpha, Small	Size 2 reaming Ø60 mm
Patient 3	Large	Large	L40	Size 3 reaming Ø80 mm	Alpha, Large	Size 3 reaming Ø80 mm
Patient 4	Small	Small	S30	Size 1 reaming Ø60 mm	Alpha, Small	Size 1 reaming Ø60 mm
Patient 5	Medium	Medium	M30	Size 2 reaming Ø80 mm	Alpha, Small	Size 2 reaming Ø80 mm
**Simulation parameters**						
* Mesh density (number of elements)*						
Glenoid component (PE)	7 400	6 400	5 600	5 900	5 900	5 800
Glenoid component (Ti)					1 650	900
Cement mantle	4 500	3 400	3 500	4 200	3 400	2 300

Abbreviations: *Ti* Titanium, *PE* Polyethylene, *P* patient, *PG* pegged-glenoid, *KG* keeled-glenoid, *CG* caged-glenoid

**Fig. 2:**
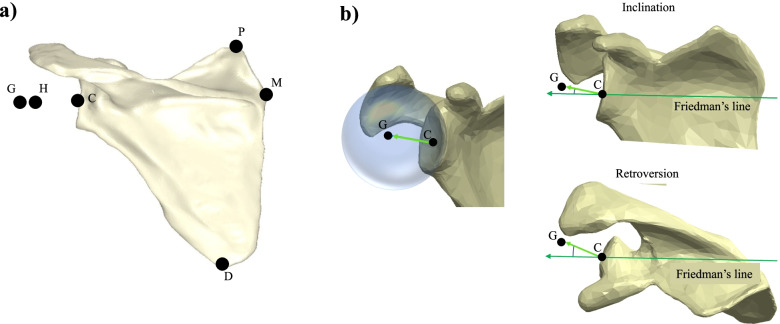
**a** Illustration of the anatomic landmarks used to define the coordinate system on each scapula. **b** Definition of glenoid version and inclination around Friedman’s line. (Abbreviations: P, most proximal point on the scapula body; M, most medial point on the medial spine; D, most distal point on the scapula body; C, glenoid center of surface (approximated through an ellipsoid); G, glenoid center of rotation (approximated through a least square spherical fit); H, humeral head center(approximated through a least squares spherical fit))

Each of the six glenoid components was virtually positioned on the 5 scapulae under the supervision of a senior surgeon (NB) following the manufacturers’ surgical guidelines, resulting in a total of 30 shoulder models. A standardized coordinate system was defined based on the anatomical landmarks (Fig. 2). Glenoid version and inclination were measured in the newly defined coordinate system, after fitting a plane to the glenoid surface by minimizing the sum of the square-of-errors between the fitted plane and points on the glenoid surface. Glenoid size was calculated following a similar approach, by fitting a sphere to the glenoid surface.

**Fig. 3:**
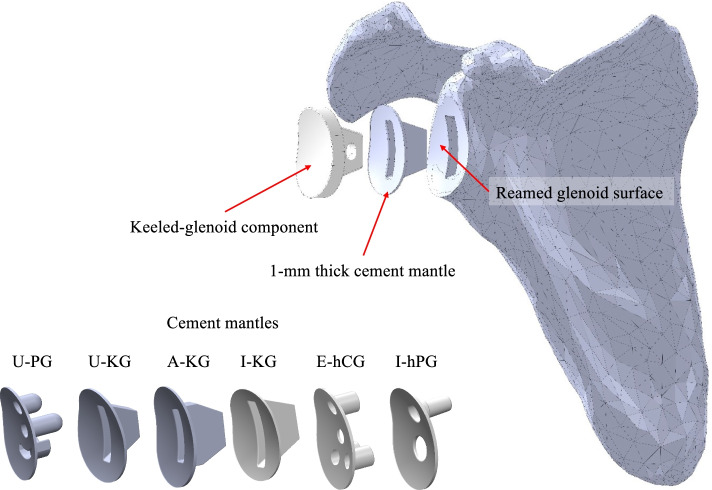
Illustration of an all-PE glenoid component, its cement mantle, and the reamed glenoid surface on the scapula

After virtual positioning, optimal bone-implant fits were created with Boolean subtract functions. Cement mantles of 1-mm thickness were applied in accordance with the manufacturer’s guidelines for component fixation (Fig. 3). All components were modeled as linear isotropic materials [7]: Polyethylene (ultra-high molecular weight polyethylene (UHMWPE); E, 361 mPa; Poisson ratio, 0.4101 m, ISO 5834–1&2); Titanium (Ti6Al4V-ELI; E, 112.4 gPa; Poisson ratio, 0.34) [10]; Cement (E, 2.0 gPa; Poisson ratio, 0.3) [3].

### Simulation

Quadratic tetrahedral volume meshes (10 nodes-per-element) were generated for 3D anatomic and implant CAD models in Solidworks 2016 (Dassault Systèmes, SolidWorks Corporation, Waltham, MA). The maximum element edge-length was 2 mm, and the mesh quality was set to a maximum of 0.1 mm difference between the 3D CAD models and the 3D mesh models. The anatomic model mesh densities (cortical and trabecular bone combined) ranged from 154 000 to 222 000 elements (Table 1), and glenoid component mesh densities (PE, Ti and cement combined) ranged from 9 000 to 11 900 elements (Table 2).

**Fig. 4:**
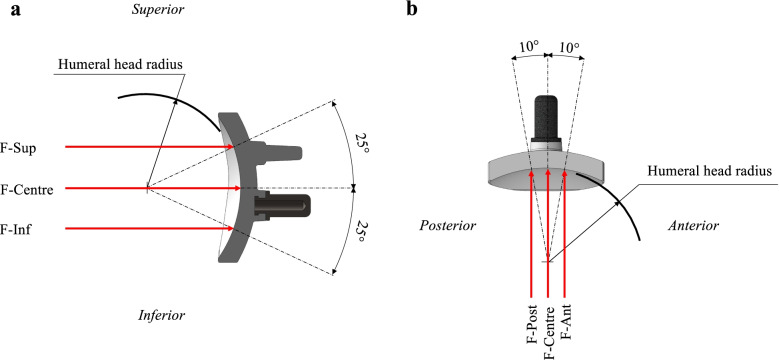
Illustration of the glenohumeral joint reaction forces. **a** Coronal view of the forces applied on the superior (F-Sup), central (F-Centre) and inferior (F-Inf) regions. **b** Transverse view of the forces applied on the anterior (F-Ant), central (F-Centre) and posterior (F-Post) regions

Glenohumeral joint reaction forces were applied at five loading regions to simulate relative movement between the humeral head and glenoid component: 1) loading on the central region of the glenoid (F-Centre); 2) loading on the anterior region of the glenoid (F-Ant); 3) loading on the posterior region of the glenoid (F-Post); 4) loading on the superior region of the glenoid (F-Sup); and 5) loading on the inferior region of the glenoid (F-Inf) (Fig. 4). The magnitudes of the joint reaction force ($F$) were related to the bodyweight of each person according to a previously proposed relation (Eq. 1) [14]:

1 $$F=0.86\times BW$$

With

$$F\;\mathrm{The}\;\mathrm{joint}\;\mathrm{reaction}\;\mathrm{force}$$

$$BW\;\mathrm{The}\;\mathrm{body}\;\mathrm{weight}\;\mathrm{of}\;\mathrm{the}\;\mathrm{patient}$$

The joint reaction forces among the five anatomic models, ranged between 404 and 903 N (Table 1), and were applied perpendicular to sagittal plane on the articulation surface of the glenoid components to simulate a worst-case scenario. The size of the contact region was based on the contact area between the prosthetic humeral head and glenoid as predicted by Hertz theory [15] (Additional file 2).

**Fig. 5:**
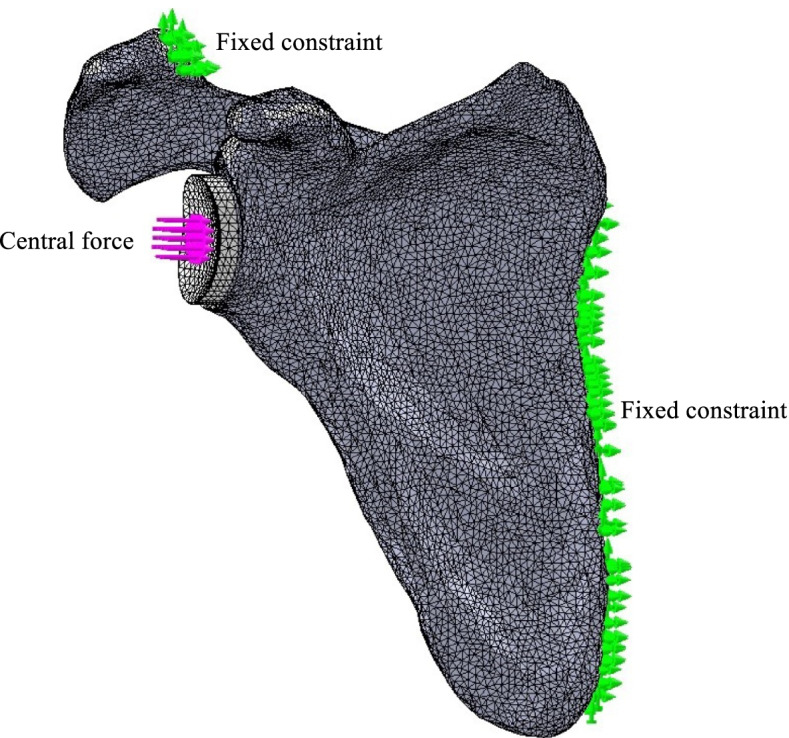
Illustration of the fixed constraints on the scapula when applying a load in the central region

Tied constraints were applied on the cortical and trabecular bone boundaries, and between the cement and cemented glenoid parts [11]. Sliding with friction and no-penetration contact constraints were applied between bone and cement (coefficient of friction, 0.6) [11, 16, 17], and between the porous structures of the hybrid components and cortical and trabecular bone (coefficient of friction, 0.74) [18]. The five scapulae were anchored at their medial aspect and at the acromial clavicular joint (Fig. 5).

The following output variables were analyzed for the 5 loading configurations on the 6 glenoid components virtually implanted in the 5 scapulae (150 sets): 1) micromotions were calculated from mean resultant nodal displacements (manual selection) in the superior-inferior, medial–lateral, and anterior–posterior directions at the bone-implant interface; 2) mean von Mises stresses within cortical and trabecular bone, and cement. Implant failure was applied according to previously suggested criteria [7]: First, implant failure was assumed to take place if glenoid micromotion exceeded a threshold of 75 µm, since studies have recommended thresholds varying between 50 μm and 150 μm [19–22]. Second, failure initiation in the cement mantle started if cement stresses exceeded 4 mPa. The critical cement volume (CCV) [7] was calculated to quantify the percentage of cement volume that exceeded the 4 mPa threshold.

The finite element analyses were done with an implicit linear static solver (Solidworks 2016, Dassault Systèmes, SolidWorks Corporation, Waltham, MA), and convergence was achieved with a displacement threshold of 1.0e-06 and the maximum number of iterations set at 1.0e + 07. All the analyses were executed on an Intel® CORE™ i7-6700 @3.40 GHz workstation equipped with 16 GB RAM and an NVIDIA Quadro K420 graphics card. Simulation wall-time durations were on average 60 min per simulation.

### Model verification

To ensure mesh independence of the predicted stresses and micromotions across simulations, the criterion for mesh convergence was defined as a change of less than 5% in the maximum displacement between mesh densities. A mesh convergence analysis on model P1 with implant I-KG, revealed that decreases in element edge lengths beyond 2 mm yielded changes < 0.05% in maximum and mean displacements. Decreases in element edge lengths beyond 2 mm yielded changes of < 8.0%, < 9.2% and < 12.7% in respectively cortical bone, trabecular bone, and cement mean von Mises stresses. Therefore, the maximum element edge lengths were limited to 2 mm in all finite element models.

### Statistical analysis

Descriptive statistics were used to summarize the data. Micromotions and stresses within bone and cement were summarized in boxplots across the five loading configurations as applied on the five anatomical models, thereby resulting in 25 results per outcome for each of the 6 glenoid components. Differences between glenoid components were evaluated using pairwise Wilcoxon Rank Sum tests with Bonferroni correction. Statistical analyses were performed with R version 3.1.1 (R Foundation for Statistical Computing, Vienna, Austria). *P* values < 0.05 were considered statistically significant.

## Results

### Cement stresses

**Fig. 6:**
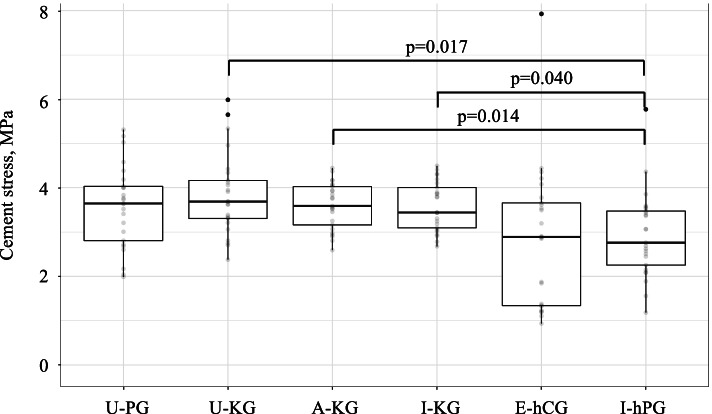
Boxplots of the pooled cement stresses among patients for each of the glenoid components

Lower average cement stresses were observed in both hybrid components compared to the all-PE pegged- and keeled-components, but pairwise comparisons only revealed statistically significant differences between the I-hPG hybrid pegged-component (2.9 ± 1.0 mPa) and the all-PE keeled components (U-KG: 3.8 ± 0.9 mPa, *p* = 0.017; A-KG: 3.6 ± 0.5 mPa, *p* = 0.014; I-KG: 3.6 ± 0.5 mPa, *p* = 0.040) (Table 3, Fig. 6).

**Table 3:** Implant micromotion, cement and bone stresses, and critical cement volume for all-polyethylene and hybrid glenoid components among all patients and load configurations

	**All-polyethylene components**																**Hybrid components**							
	**U-PG**				**U-KG**				**A-KG**				**I-KG**				**E-hCG**				**I-hPG**			
	mean	± SD	(range)		mean	± SD	(range)		mean	± SD	(range)		mean	± SD	(range)		mean	± SD	(range)		mean	± SD	(range)	
**Stresses**																								
Cement stress (MPa)	3.6	± 0.9	(2.0	– 5.3)	3.8	± 0.9	(2.4	– 6.0)	3.6	± 0.5	(2.6	– 4.4)	3.6	± 0.6	(2.7	– 4.5)	2.8	± 1.6	(0.9	– 7.9)	2.9	± 1.0	(1.2	– 5.8)
Cortical bone (MPa)	1.8	± 0.3	(1.4	– 2.5)	1.8	± 0.3	(1.1	– 2.6)	1.8	± 0.3	(1.4	– 2.6)	1.8	± 0.4	(1.2	– 2.7)	1.8	± 0.3	(1.4	– 2.5)	1.7	± 0.3	(1.2	– 2.5)
Trabecular bone (MPa)	0.8	± 0.2	(0.4	– 1.1)	0.8	± 0.2	(0.4	– 1.1)	0.8	± 0.2	(0.4	– 1.3)	0.8	± 0.2	(0.4	– 1.1)	0.8	± 0.2	(0.5	– 1.4)	0.7	± 0.2	(0.3	– 1.0)
**Micromotion**																								
Glenoid micromotion (µm)	11.6	± 12.3	(0.2	– 48.7)	11.1	± 11.2	(0.4	– 43.5)	8.6	± 8.6	(0.2	– 45.0)	12.5	± 11.0	(0.8	– 59.7)	12.9	± 15.3	(0.4	– 93.8)	7.9	± 7.4	(0.5	– 33.2)
**Failure analysis**																								
%CCV (> 4 MPa)	39.6	± 7.4	(25	– 53)	43.0	± 10.5	(25	– 59)	42.5	± 12.2	(20	– 63)	44.5	± 13.5	(21	– 66)	32.5	± 17.0	(8	– 65)	34.8	± 14.8	(6	– 68)

Abbreviations: *PG* pegged-glenoid, *KG* keeled-glenoid, *CG* caged-glenoid, *SD* standard deviation, *CCV* critical cement volume

### Bone stresses

**Fig. 7:**
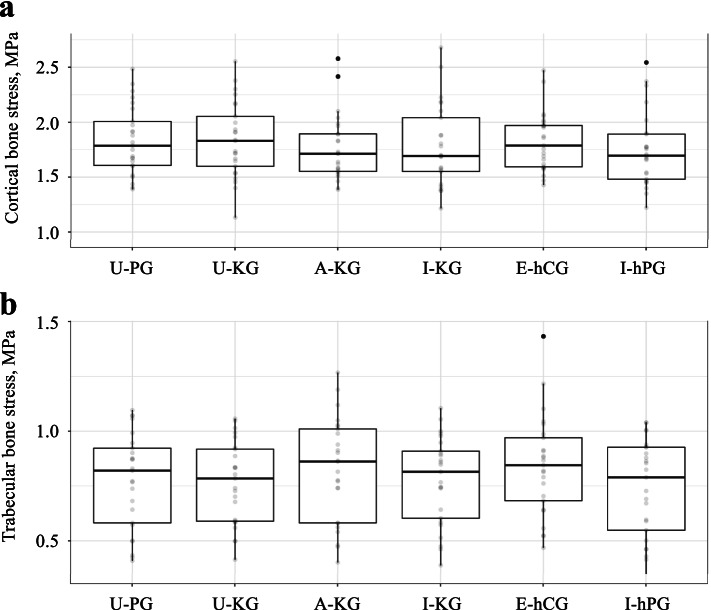
Boxplots of the pooled bone stresses among patients for each of the glenoid components. **a** Cortical bone stresses. **b** Trabecular bone stresses

No significant differences in stresses within cortical (range, 1.7 to 1.8 mPa) and trabecular bone (range, 0.7 to 0.8 mPa) were observed among the different glenoid components (Table 3, Fig. 7).

### Micromotion

**Fig. 8:**
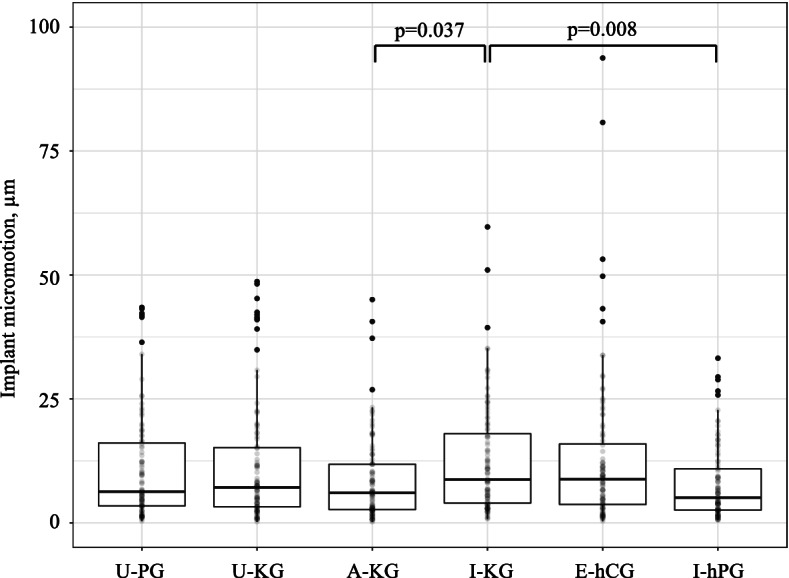
Boxplots of the pooled implant micromotions among patients for each of the glenoid components

Micromotions were significantly higher using the I-KG all-PE keeled-component (12.5 ± 11.0 µm), compared to the A-KG all-PE keeled-component (8.6 ± 8.6 µm, *p* = 0.037) and I-hPG hybrid pegged-component (7.9 ± 7.4 µm, *p* = 0.008) (Table 3, Fig. 8).

### Failure analysis

**Fig. 9:**
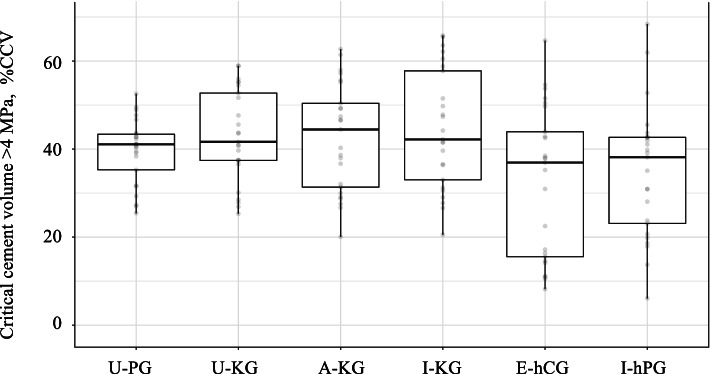
Boxplots of the pooled critical cement volume (%CCV) among patients for each of the glenoid components

For all patients and loading configurations, only the E-hCG hybrid caged-component exceeded the implant micromotion threshold of 75 µm on 2 occasions: First, the maximum displacement was 93.8 µm for an anterior joint reaction force in the model of Patient 1; and second, the maximum displacement was 80.8 µm for a central joint reaction force in the model of Patient 3. For all patients and loading configurations, no statistically significant differences were observed among the different components (Table 3, Fig. 9).

## Discussion

The most important finding of this study was that compared to all-PE glenoid components, hybrid components yielded similar average stresses within bone and cement, except for one hybrid component that yielded significantly lower average cement stresses. Moreover, no differences were observed for %CCV between the hybrid and all-PE glenoid components. In addition, micromotions were less than 75 µm for all components, except for two instances when using one of the hybrid components. These findings therefore only partly support the hypothesis of lower cement stresses, and refutes the hypotheses of lower bone stresses and smaller micromotions when using hybrid compared to all-PE glenoid components.

Cemented all-PE components have lower revision rates in comparison to uncemented components, but when comparing rates of loosening or lysis at a follow-up of 5 years, there is no difference between cemented and uncemented components (1.1%) [8]. Although the cemented all-PE component is associated with adequate initial fixation and stability [23], fatigue can lead to micro-cracks and ultimate failure of the cemented fixation [7]. Therefore, the lower stresses in cement when using hybrid components are advantageous since it may lead to reduced risks of cement overloading. The findings of the present study also revealed no difference in stresses in bone among the cemented all-PE and hybrid glenoid components, which is encouraging, since excessive early loading will result in poor primary stability prior to osseointegration. Consistent mechanical loading in implants with sufficient primary stability is critical to bone tissue formation and maintenance [24].

Excessive micromotion and strains can stimulate fibrosis on the implant surface and negatively impact bone-implant stability [24]. In the present study, micromotions exceeded the 75 µm threshold in only two instances for one of the hybrid components; however, the threshold of 75 µm can be considered conservative, since studies have recommended thresholds varying between 50 μm and 150 μm [19–22]. Even though the implants were exposed to substantial loading conditions, micromotion remained below 150 µm for all simulations. Therefore, the results revealed favorable conditions that will promote osseous-integration within the porous Ti components, irrespective of the osseointegration kinetics.

A recent systematic review showed that modern metal-backed glenoid components have improved survival rates compared to conventional metal-backed components [1]. Since these implants offer the potential of long-term stability through biologic fixation, they are considered promising alternatives to cemented all-PE components. Their success depends on sufficient initial stability to promote osseointegration, which can be problematic in patients with poor bone quality [7]. The mean %CCV in the present study ranged from 32.5% to 44.5% for all components, indicating possible failure due to fatigue. As hybrid components only rely on cemented fixation for initial stability, it may be less prone to fatigue failure as biological fixation becomes the major contributor to implant stability after approximately 3 months [25].

The results of this study should be considered with the following limitations in mind. First, although the models account for variations in joint reaction force magnitude and bone properties of cortical and cancellous bone, the impact of soft tissue tensions and conditions was not considered. Other time-dependent biological factors, such as osseous-integration, were not considered in the analysis. Second, the cohort of 5 scapulae could be considered small, and therefore may not represent the general population. Third, bone mineral density was based on CT Hounsfield Units, and the CT scanner settings were not optimized with a calibration phantom. The scans for each patient were however obtained with the same CT scanner, of which scanning parameters and post-processing settings were standardized and consistently applied. Fourth, the present study only compared all-PE components to hybrid components and did not include metal-backed components. It would be interesting to include metal-backed components in future comparative analyses, since a recent clinical study of 37 TSA with metal-backed glenoid components found 100% implant survival at mean 7-year follow-up, despite the occurrence of metallic debris formation [25]. Fifth, quasi-static loads were applied to the glenoid components, and neither fatigue failure nor micromotion and stresses could be verified against in-vivo measurements. Moreover, reasons for the observed variation in average cement stresses among the baseplates could be multifactorial, such as differences in morphology, i.e., pegs versus fins versus a combination of pegs and fins, or the cementing technique. Finally, there are differences in the reaming process which is not accounted for in the FEA: For the glenoid models with pegs, a reaming is performed which mainly removes bone, whereas for the keel, after reaming the small holes, an impact process is needed for completing the hole, which could lead to differences on the underlying trabecular bone.

## Conclusion

Finite element analyses reveal that compared to all-PE glenoid components, hybrid components yield similar average stresses within bone and cement. Finally, risk of fatigue failure of the cement mantle is equal for hybrid and all-PE components, as no difference in %CCV was observed.

## Supplementary Information

**Additional file 1.** Anatomical material model.

**Additional file 2.** Estimation of contact area.
