# Supplementary material for: Mechanical behavior of hybrid glenoid components compared to all-PE components: a finite element analysis
Source: J Exp Orthop. 2022 Jun 19;9:58. doi: 10.1186/s40634-022-00494-8 (PMC9206973; doi:10.1186/s40634-022-00494-8)
Supplement: Supplementary file 1 — Additional file 1. Anatomical material model. [file 40634_2022_494_MOESM1_ESM.docx]

**Additional file 1: Anatomical material model**

The bone density in each voxel was estimated by applying the method described by Pomwenger et al [15] (eq. A.1)

| $\rho_{i}=k\times\rho_{HU}$ | (A.1) |
| --- | --- |
| With |  |
| $\rho_{i}$ | Bone density in $g/{cm}^{3}$ per voxel. |
| $k$ | A subject-specific scaling coefficient |
| $\rho_{HU}$ | The Hounsfield unit value |
| $i$ | Current voxel number |

The subject-specific scaling coefficient ($k$), was calculated for each scapula model using (eq. A.2):

| $k=\rho_{max}\times\rho_{HU, max}$ | (A.2) |
| --- | --- |
| With |  |
| $\rho_{max}$ | 1.8 $g.{cm}^{-3}$ |
| $\rho_{HU,max}$ | The maximum Hounsfield unit value |

Finally, mean cortical ($\bar{\rho}_{cortical}$) and mean trabecular ($\bar{\rho}_{trabecular}$) densities were first calculated (eq. A.3), before the Young’s moduli ($E$) were assigned to the cortical and trabecular bone volumes according to the method proposed by Pomwenger et al. [14] (eq. A.4). A Poisson ratio of 0.3 was assigned for both cortical and trabecular bone [3].

| $\bar{\rho}_{i,j}=\frac{\sum_{i=1}^{n_{j}} \rho_{i,j}}{n_{j}}$ | (A.3) |
| --- | --- |
| With |  |
| $j$ | Cortical or trabecular bone in the current CT slice. |
| $i$ | Current voxel number in the current CT slice. |
| $n_{j}$ | Total number of voxels in the current CT slice. |

| $E=\left\{ \begin{matrix} 3000\times\bar{\rho}^{3} & 0.35 g.{cm}^{-3}\leq\bar{\rho}\leq1.8 g.{cm}^{-3} \\ 1049.45\times\bar{\rho}^{2} & \bar{\rho}<0.35 g.{cm}^{-3} \end{matrix} \right.$ | (A.4) |
| --- | --- |
