# Supplementary material for: Mechanical behavior of hybrid glenoid components compared to all-PE components: a finite element analysis
Source: J Exp Orthop. 2022 Jun 19;9:58. doi: 10.1186/s40634-022-00494-8 (PMC9206973; doi:10.1186/s40634-022-00494-8)
Supplement: Supplementary file 2 — Additional file 2. Estimation of contact area. [file 40634_2022_494_MOESM2_ESM.docx]

**Additional file 2: Estimation of contact area**

The size of the contact region was based on the contact area between the prosthetic humeral head and glenoid as predicted by Hertz theory (eq. B.1) [17]:

| $a=\sqrt[3]{\frac{3F\left( \frac{R_{1}R_{2}}{R_{2}-R_{1}} \right)}{4E^{*}}}$ | (B.1) |
| --- | --- |
| With |  |
| $a$ | Radius of the contact area. |
| $F$ | Joint reaction force. |
| $R_{1}$ | Radius of the humeral head. |
| $R_{2}$ | Radius of the glenoid surface. |
| $E^{*}$ | The equivalent Young’s modulus. |

The equivalent Young’s modulus was based on (eq. B.2):

| $E^{*}=\frac{1-\upsilon_{1}^{2}}{E_{1}}+\frac{1-\upsilon_{2}^{2}}{E_{2}}$ | (B.2) |
| --- | --- |
| With |  |
| $E_{1}$ | Young’s modulus of glenoid material in contact. |
| $E_{2}$ | Young’s modulus of humeral head material in contact. |
| $\upsilon_{1}$ | Poisson ratio of glenoid material in contact. |
| $\upsilon_{2}$ | Poisson ratio of humeral head material in contact. |
